# Body image in idiopathic scoliosis: a comparison study of psychometric properties between four patient-reported outcome instruments

**DOI:** 10.1186/1748-7161-9-S1-O74

**Published:** 2014-12-04

**Authors:** Elisabetta DAgata, Antonia Matamalas, Joan Bago, Ferran Pellise

**Affiliations:** 1 Fundacion Hospital Vall Hebron, Barcelona, Spain; 2 Hospital Vall Hebron, Barcelona, Spain

## Background

Four patient-reported outcome (PRO) instruments are used to assess body image in idiopathic scoliosis (IS): Quality of Life Profile for Spinal Deformities (QLPSD), SRS-22 Self Image scale, Spinal Appearance Questionnaire (SAQ), and Trunk Appearance Perception Scale (TAPS).

## Aim

To compare the psychometric properties of these four assessment instruments.

Design: This is a cross-sectional study. Inclusion criteria were patients with IS, 10 to 40 years old, Cobb angle ≥25°, without previous surgical treatment..

## Methods

80 patients (mean age 20.3 years) were included. The four instruments in a Spanish version were administered. In addition, full-spine x-ray was obtained. Sample was stratified into two groups according to Cobb angle (less and more than 45º). Psychometric properties studied included internal consistency, convergent (correlation between self-image scales and Cobb angle) and divergent validity (correlations with Health Related Quality of Life domains: function, pain, mental health, measured through SRS-22).

## Results

All the PRO instruments presented high internal consistency (QLPSD Body Image, α=0.80; SRS-22 Image, α=0.78; SAQ, α=0.89; TAPS, α=0.87).Pictorial scales showed higher correlations with Cobb angle (SAQ Appearance r=0.61 and TAPS r=-0.62) than textual scales (QLPSD-bi (r=0.36; SRS-22 Self-Image r=-0.41).The four image scales showed significant correlations with other HRQL SRS22 dimensions (from r=-.2 to 0.7).

## Conclusions

All four instruments have good psychometric properties. To evaluate patients with IS is advisable to add pictorial image perception scales to HRQL assessment instruments.

